# An α-hemoglobin-derived peptide (m)VD-hemopressin (α) promotes NREM sleep via the CB_1_ cannabinoid receptor

**DOI:** 10.3389/fphar.2023.1213215

**Published:** 2023-06-30

**Authors:** Jun-Fan Xie, Lin-Xin Wang, Wen-Ting Ren, Can Wang, Jin-Xian Gao, Hai-Lin Chen, Xue-Qi Zhao, Yan-Li Ren, Yu-Ping Xie, Yu-Feng Shao, Yi-Ping Hou

**Affiliations:** ^1^ Departments of Neuroscience, Anatomy, Histology, and Embryology, Key Laboratory of Preclinical Study for New Drugs of Gansu Province, School of Basic Medical Sciences, Lanzhou University, Lanzhou, China; ^2^ Departments of Anatomy, Histology, and Embryology, School of Basic Medical Sciences, Gansu University of Chinese Medicine, Lanzhou, China; ^3^ Departments of Anatomy, School of Basic Medical Sciences, Weifang Medical University, Weifang, China; ^4^ Sleep Medicine Center of Gansu Provincial Hospital, Lanzhou, China

**Keywords:** mouse VD-hemopressin (α), endocannabinoid, cannabinoid receptors, cannabinoid receptors antagonist, c-Fos, sleep–wake states

## Abstract

Hemopressin and related peptides have shown to function as the endogenous ligands or the regulator of cannabinoid receptors. The previous studies demonstrated that the endocannabinoid system played important roles in modulating several physiological functions such as sleep, olfaction, emotion, learning and memory, and reward behaviors. Mouse VD-hemopressin (α) [(m)VD-HPα], an 11-residue peptide derived from the α1 chain of hemoglobin, was recently presumed as a selective agonist of the CB_1_ receptor. The present study was undertaken to investigate the effects of (m)VD-HPα on the sleep–wake cycle and power spectrum of cortical EEG in freely moving rats and the potential neurons in the brain activated by (m)VD-HPα. The results showed that 20.1 nmol of (m)VD-HPα i.c.v. administration increased non-rapid eye movement (NREM) sleep in the first 2 h section accompanied by an increase in EEG delta (0.5–4 Hz) activity. The (m)VD-HPα-induced NREM sleep enhancement was due to extended episode duration instead of the episode number. In addition, the effect of (m)VD-HPα (20.1 nmol) on sleep–wake states was significantly attenuated by an antagonist of the CB_1_ receptor, AM251 (20 nmol, i.c.v.) but not by the CB_2_ receptor antagonist, AM630 (20 nmol, i.c.v.). In comparison with vehicle, (m)VD-HPα increased Fos-immunoreactive (-ir) neurons in the ventrolateral preoptic nucleus (VLPO), but reduced Fos-ir neurons in the lateral hypothalamus (LH), tuberomammillary nucleus (TMN), and locus coeruleus (LC). These findings suggest that (m)VD-HPα promotes NREM sleep via the CB_1_ cannabinoid receptor to probably activate VLPO GABAergic neurons, but inactivates the LH orexinergic, LC noradrenergic, and TMN histaminergic neurons.

## 1 Introduction

Cannabinoids, including synthetic cannabinoids, phytocannabinoids, and endocannabinoids, exert their biological effects by acting on cannabinoid receptors (Mendiguren and Pineda, 2006; Pacher et al., 2006). Two known types of cannabinoid receptors, termed cannabinoid type 1 (CB_1_) and 2 (CB_2_) receptors, have been characterized and cloned (Svízenská et al., 2008). The CB_1_ receptor is widely distributed in the brain, mainly in the olfactory bulb, hippocampus, cerebral cortex, amygdala, hypothalamus, accumbens nucleus, and parts of the brainstem (Svízenská et al., 2008; Zou and Kumar, 2018). The CB_2_ receptor is located principally in immune cells, having a lower level of expression in the brain (Liu et al., 2009; Zou and Kumar, 2018). Endocannabinoids, cannabinoid receptors, and the whole machinery for the synthesis and degradation of endocannabinoids form the endocannabinoid system (Monory and Lutz, 2009), which has emerged as an important system in controlling numerous physiological processes, including learning and memory, reward, feeding, anxiety, depression, and sleep–wake cycle (Zuardi, 2008; Monory and Lutz, 2009; Zanelati et al., 2010; Bergamaschi et al., 2011; Chagas et al., 2013). The endocannabinoid system is traditionally considered to be modulated by lipophilic endocannabinoids. However, recent research has demonstrated that endocannabinoid receptors are also regulated by hemopressin peptides (Heimann et al., 2007; Gomes et al., 2009; Han et al., 2014). Hemopressin, a nonapeptide derived from the α1-chain of hemoglobin (Dale et al., 2005; Han et al., 2014), was reported to function as an endogenous inverse agonist or antagonist of the cannabinoid receptors (Heimann et al., 2007; Dodd et al., 2010). Subsequently, a related peptide of hemopressin, mouse VD-hemopressin(α) [(m)VD-Hpα], was identified in mouse brain extracts (Gomes et al., 2009). Compared with hemopressin, (m)VD-Hpα showed agonistic activity at cannabinoid receptors, particularly, selectively activated CB_1_ receptors (Gomes et al., 2009).

The regulation of the sleep–wake cycle is complex and involves multiple neuroanatomical nuclei and diverse endogenous molecules (Siegel, 2004; Jones, 2005; Saper et al., 2005), such as norepinephrine, histamine, orexin, γ-aminobutyric acid (GABA), and neuropeptide S (Jones, 2005; Zhao et al., 2012; Xie et al., 2018). A breadth of evidence has demonstrated that the endocannabinoid system plays a critical role in regulating the sleep–wake cycle (Huitron-Resendiz et al., 2004; Prospéro-García et al., 2016). Indeed, administration of anandamide facilitates rapid eye movement (REM) and non-REM (NREM) sleep through activating the CB_1_ receptor (Murillo-Rodríguez et al., 2001; Rueda-Orozco et al., 2010), and mice lacking fatty acidamide hydrolase, the enzyme responsible for hydrolyzing anandamide, have been shown increased sleep and reduced wakefulness (Huitron-Resendiz et al., 2004). Systemic administration of CB_1_ receptor antagonist SR141716A increases wakefulness in rats (Santucci et al., 1996). The sleep–wake profiles of several non-peptidic agonists of cannabinoid receptors have been well-investigated; however, the effects of the endogenous peptidic agonists of the cannabinoid system on the sleep–wake state have not been characterized. Thus, further study of (m)VD-Hpα and other hemopressin-related peptides could be helpful to characterize the role of the endocannabinoid system on the sleep–wake cycle.

Therefore, the present study was designed to investigate the effects of intracerebroventricular (i.c.v.) administration of (m)VD-HPα on the sleep–wake cycle. Attempts were also made to identify the potential neuronal targets of (m)VD-HPα in the ventrolateral preoptic nucleus (VLPO), lateral hypothalamus (LH), tuberomammillary nucleus (TMN), and locus coeruleus (LC) involved in the sleep–wake regulation by analyzing c-Fos expression using *ex vivo* Fos immunohistochemistry.

## 2 Materials and methods

### 2.1 Animals and surgical implantation

Adult male Sprague–Dawley rats (8–10 weeks old), weighing 250–300 g, were purchased from the Experimental Animal Center of Lanzhou University (Lanzhou, PR China). Upon arrival at the animal housing facility, they were kept in an automatically controlled room on a 12:12-h light/dark cycle (lights on 8:00–20:00 h, illumination intensity ≈100 lux) at an ambient temperature (22°C ± 1°C) and 50% relative humidity with food and water available *ad libitum.* All animals were cared for, and experiments were conducted in accordance with the National Institutes of Health Guide for the Care and Use of Laboratory Animals (2011 revision). The experimental protocol was approved by the Ethics Committee of Lanzhou University (permit number: SCXK Gan 2018-0002). All possible efforts were made to reduce the number of rats used and discomfort to the rats.

Under pentobarbital sodium anesthesia (50 mg/kg, i.p.), four stainless steel screw cortical electrodes (1 mm diameter) were screwed through the skull into frontal (2 mm lateral and anterior to the bregma) and parietal (2 mm lateral to the lambda) cortices to record electroencephalogram (EEG). Three silver wires were inserted into the dorsal cervical neck muscles to record electromyogram (EMG). The free ends of the electrode leads and silver wires were soldered into a seven-pin miniature plug. For i.c.v. injection, a guide cannula (0.6 mm diameter and 20 mm long) was stereotaxically implanted into the right lateral ventricle (AP −0.9, ML +1.5, DV −3.3) of the rat according to the atlases of Paxinos and Watson (2007). The plug and cannula were chronically fixed to skull with dental cement. After surgeries, rats were allowed to recover for 1 week.

### 2.2 Drug administration

(m)VD-HPα (VDPVNFKLLSH) was synthesized by Shanghai Mocell Biotech Co., Ltd., Shanghai, PR China. Fresh (m)VD-HPα (6.7, 13.4, and 20.1 nmol), Win55,212-2 (2.5 nmol; Sigma-Aldrich), AM251 (20 nmol; Tocris), and AM630 (20 nmol; MedChemExpress) were dissolved in a 5-μL vehicle containing 5% cremophor, 5% dimethyl sulfoxide, and 90% saline before use according to Han et al. (2014). The drugs or equal volume of vehicle were administrated into the lateral ventricle through the planted guide cannula at a speed of 1 μL/min 5 min prior to sleep–wake recording at 20:00 h (Zhao et al., 2012; Shao et al., 2016; Xie et al., 2017; Xie et al., 2018).

After the experiments, 5 μL of methylene blue dye was injected into the ventricle via the guide cannula 5 min before the rats were decapitated under deep anesthesia with chloral hydrate. The brains were removed and frozen. Gross dissection of the brain was used to verify the site of drugs and vehicle administration; two rats whose microinjection sites were not located within the i.c.v. were excluded. Only the data from animals with dye dispersion throughout the ventricle were used.

### 2.3 Polygraphic recordings and sleep–wake states analysis

After surgery, the rats were housed singly in transparent barrels (Φ = 300 mm, height = 400 mm) and monitored using an infrared video camera in the recording chamber during both light and dark phases, and they were allowed to recover for 1 week. After the rats were connected to the recording cable attached to a slip ring for a habituation period of 2 days, a 24-h sleep–wake recording (20:00–20:00 h) was performed.

EEG and EMG activities were amplified (2000×), filtered (0.5–40 Hz for EEG and 30–300 Hz for EMG, Model 3500, A-M Systems, WA, United States), and digitalized at a resolution of 256 and 128 Hz, respectively, using CED 1401 MK II [Cambridge Electronic Design Limited (CED), London, United Kingdom]. The behaviors of the rats during the light and dark phases in the chamber were monitored and recorded using an infrared video camera. Using a Spike 2 (CED) sleepscore script and with the assistance of spectral analysis by the fast Fourier transform (FFT), we visually scored polygraphic records by 30-s epochs for wakefulness (W), REM, and NREM sleep according to our previously described criteria validated for rats (Zhao et al., 2012; Xie et al., 2017; Xie et al., 2018).

### 2.4 Immunohistochemistry

#### 2.4.1 Tissue preparation

After 90 min of (m)VD-HPα (20.1 nmol, *n* = 5) or vehicle (*n* = 5) i.c.v. administration, the rats were deeply anesthetized with pentobarbital sodium and perfused via the ascending aorta with 200 mL saline followed by 300 mL ice-cold 4% paraformaldehyde (PFA) in 0.1 M phosphate buffer (PB, pH 7.4). The brains were removed, post-fixed in 4% PFA overnight, immersed in 30% sucrose solution in 0.1 M PB at 4°C for 48 h, and then coronally sectioned (30 μm) on a cryostat (Thermo Scientific, United States) at −20°C.

#### 2.4.2 Fos immunostaining

The floating sections were rinsed in 0.01 M PB saline (PBS, pH 7.4), processed for 30 min in 0.3% H_2_O_2_ in PBS, and incubated in blocking buffer (10% bovine serum in PBS) for 1 h. Then, the sections were incubated with a rabbit polyclonal antibody against c-Fos (1:6000, sc-253, Santa Cruz Biotechnology, Santa Cruz, United States) in PBS containing 1% bovine serum for 72 h at 4°C on an agitator. After rinsing in PBS, the sections were incubated with a biotinylated goat anti-rabbit IgG (1:1000, AP132B, Millipore, Temecula, CA, United States) for 48 h at 4°C and then with horseradish peroxidase-conjugated streptavidin (1:2000, SA202, Millipore, Temecula, CA, United States) for 24 h at 4°C. Following rinsing, the sections were immersed in 0.05 M Tris-HCl buffer (pH 7.6), containing 0.05% 3,3’ diaminobenzidine (DAB), 0.01% H_2_O_2_, and 0.6% nickel ammonium sulfate for 2–5 min at room temperature. The sections were mounted on gelatin-coated glass slides, dried, dehydrated, and covered with a coverslip, using DPX, for light microscopy.

### 2.5 Data analysis

#### 2.5.1 Cell counting

Sections containing the VLPO (Bregma 0.00, −0.12, −0.24, −0.36), LH (Bregma −1.56, −1.80, −2.04, −2.28), TMN (Bregma −3.72, −3.96, −4.20, −4.44), and LC (Bregma −9.60, −9.72, −9.84, −9.96) were determined by the characteristics of their cytoarchitecture and peripheral white matter according to the rat atlases of Paxinos and Watson (2007). Fos-ir neurons were bilaterally counted with a counting tool of the ZEISS Efficient Navigation (ZEN) microscope software (Germany). The average of numbers from two sides of each rat was calculated and used for analysis.

#### 2.5.2 Statistical analysis

All data were expressed as means ± SEM. Statistical significance was analyzed by two-way repeated-measures analysis of variance (ANOVA) or one-way ANOVA followed by a *post hoc* Fisher’s least significant difference (LSD) test for sleep parameters and unpaired Student’s t-test for the number of Fos-ir neurons. The significance was set at *p* < 0.05.

## 3 Results

### 3.1 Effect of (m)VD-HPα on sleep–wake states and cortical EEG

**FIGURE 1:**
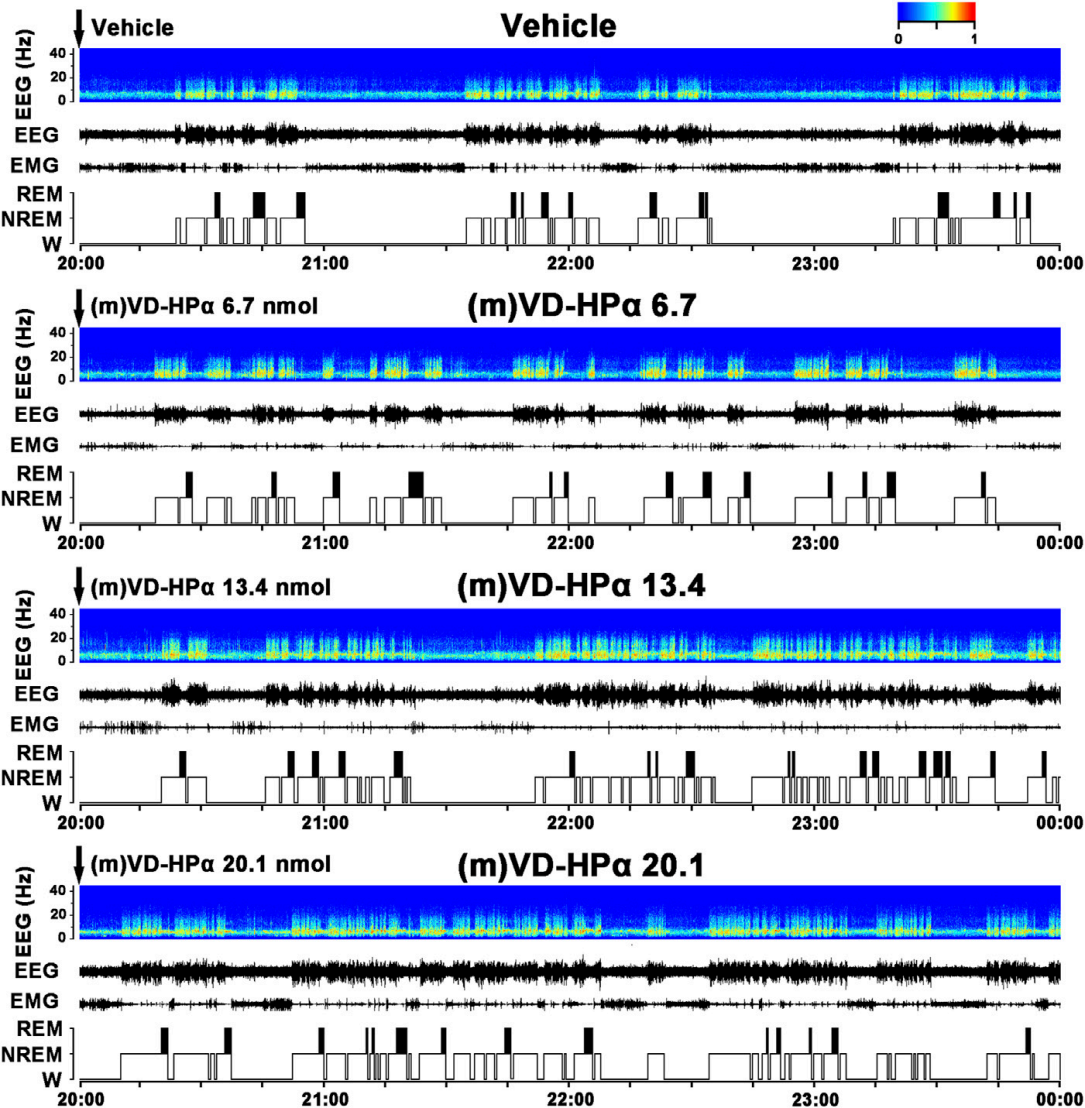
Effects of (m)VD-HPα i.c.v. injection on EEG and sleep–wake states. Representative 4-h (20:00–00:00) EEG power spectra (frequency 0–30 Hz), EEG, electromyogram (EMG), and hypnograms in rats treated with vehicle, 6.7, 13.4, and 20.1 nmol of (m)VD-HPα are shown. It should be noted that (m)VD-HPα (20.1 nmol) reduced latency to NREM sleep and induced an increase in NREM sleep accompanied by increased cortical 0.5–4.0 Hz activities and suppressed cortical 14.5–30 Hz activities.

Typical examples of the EEG power spectra, EEG, EMG, and the corresponding hypnograms from rats, respectively, given vehicle, 6.7, 13.4, and 20.1 nmol of (m)VD-HPα were presented in Figure 1. As the examples shown, i.c.v. administration of 20.1 nmol of (m)VD-HPα increased NREM sleep accompanied by slow high-voltage activity in cortical EEG and low-EMG activity compared to vehicle (Figure 1). The effects lasted 2 h and then the recovery of sleep–wake states and cortical EEG bands occurred from the third hour (Figure 1). Then, 13.4 nmol of (m)VD-HPα caused an increase in NREM sleep and delta activities during 23:00–24:00 h (Figure 1). 6.7 nmol of (m)VD-HPα did not induce any significant alteration in sleep–wake states and EEG power spectra compared with the vehicle group (Figure 1).

**FIGURE 2:**
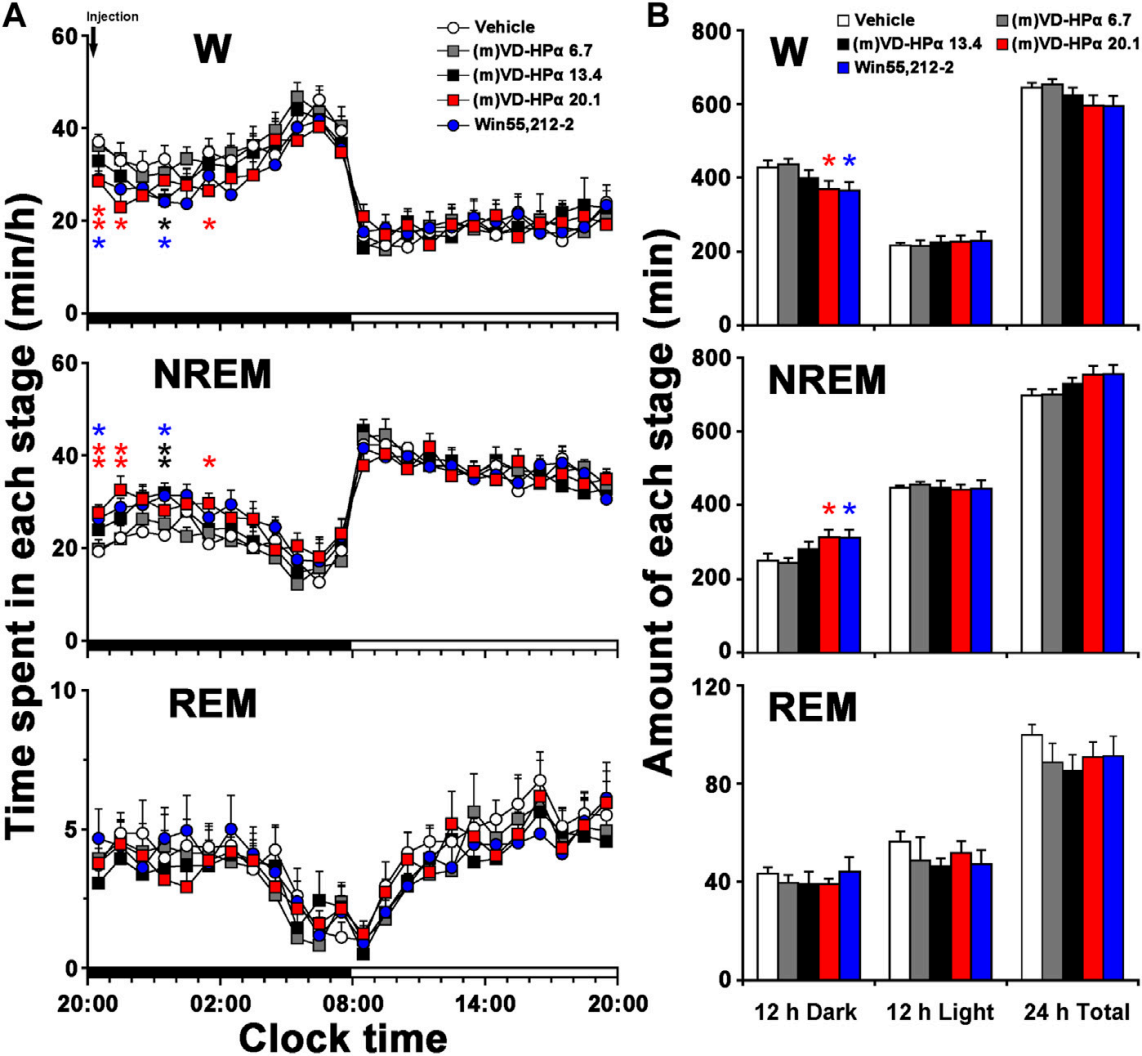
Sleep–wake profiles of rats treated with vehicle, 6.7, 13.4, and 20.1 nmol of (m)VD-HPα and Win55,212-2. **(A)** Time–course changes in wakefulness (W), NREM, and REM sleep for 24 h (*n* = 8–11, two-way repeated-measure ANOVA followed by the LSD *post hoc* test; W: F_4,41_ = 2.748, *p* = 0.041; NREM: F_4,41_ = 3.020, *p* = 0.028; and REM: F_4,41_ = 0.431, *p* = 0.785; *p < 0.05 and **p < 0.01 compared to vehicle). The horizontal filled and open bars indicate the 12-h dark and 12-h light periods, respectively. **(B)** Total time spent in W, NREM, and REM sleep during the 12-h dark and 12-h light phases and total 24 h (*n* = 8–11; one-way ANOVA followed by the LSD *post hoc* test, *p < 0.05, compared to vehicle). Data are the means ± SEM.

An analysis of the hourly amount of the time spent in each stage over 24 h revealed that compared with vehicle rats, the rat injection with 20.1 nmol of (m)VD-HPα showed an increase in NREM sleep mainly during 20:00–22:00 h and 01:00–02:00 h of clock time in the dark period (Figure 2A, *p* < 0.01, *p* < 0.01, and *p* < 0.05) and with a concomitant decrease in W (Figure 2A, *p* < 0.01, *p* < 0.05, and *p* < 0.05). Compared with vehicle, 13.4 nmol of (m)VD-HPα resulted in an increase in NREM sleep by 11.1% (*p* = 0.114), 9.8% (*p* = 0.291), 18.4% (*p* = 0.061), and 26.7% (*p* < 0.01) during 20:00–24:00 h (Figure 2A). An analysis of the amount of each stage in 12-h dark and light phases and 24-h total showed that 20.1 nmol of (m)VD-HPα increased NREM sleep by 24.8% (Figure 2B middle, *p* < 0.05), meanwhile, decreased W by 13.6% in the 12-h dark phase (Figure 2B upper, *p* < 0.05). The rat injection with 13.4 nmol of (m)VD-HPα had a 12.8% increase in NREM sleep (Figure 2B middle, *p* = 0.226) and a 6.5% decrease in wakefulness during the 12-h dark phase (Figure 2B upper, *p* = 0.319). Compared with vehicle, Win55,212-2 (2.5 nmol i.c.v.), a classic agonist of the CB_1_ receptor used as positive control, induced an increase in NREM sleep mainly during 20:00–21:00 h and 01:00–02:00 h of clock time and increased the amount of NREM sleep by 24.3% in the 12-h dark phase, which were similar to that induced by 20.1 nmol of (m)VD-HPα (Figure 2).

**FIGURE 3:**
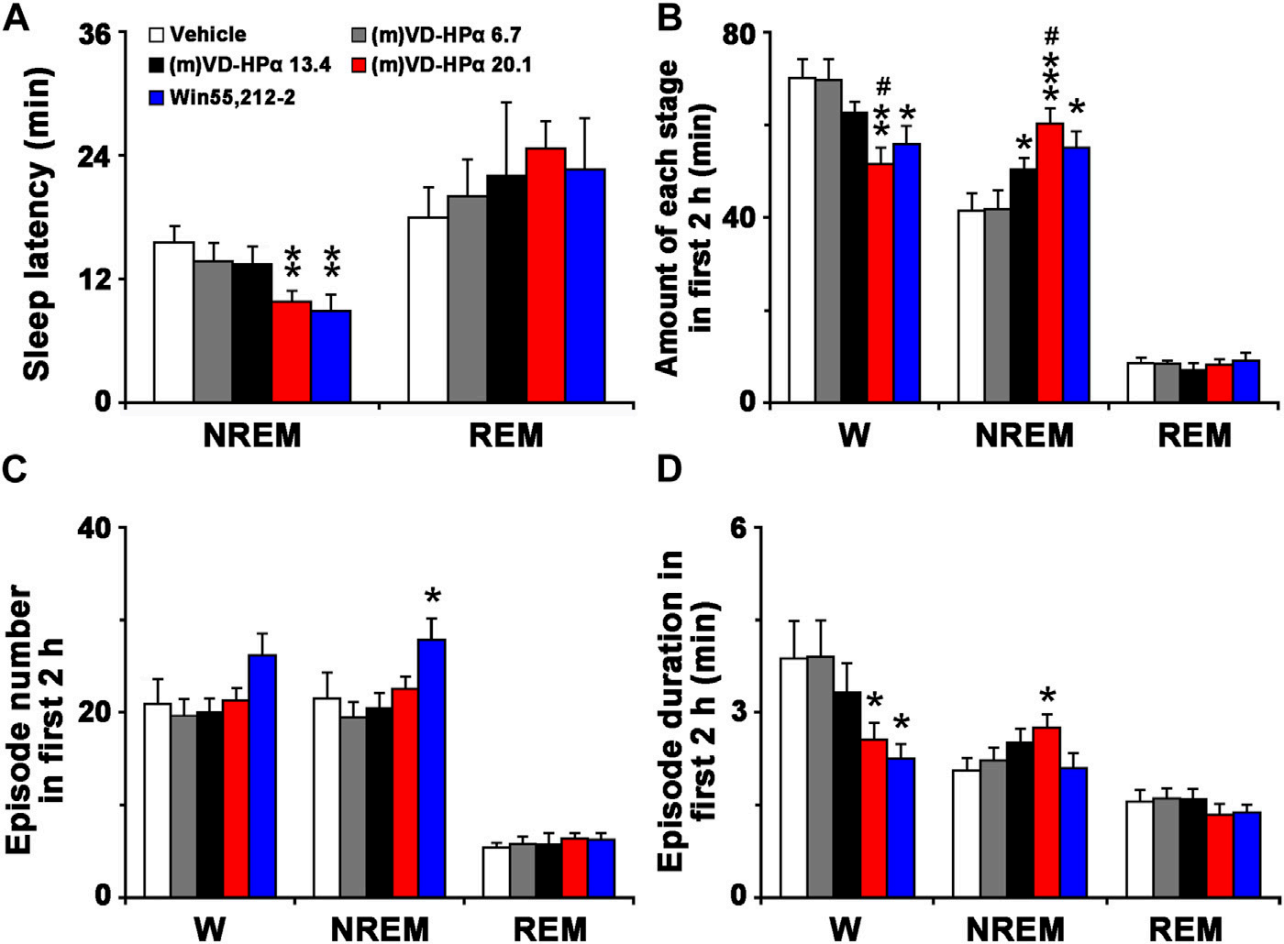
Sleep latency **(A)**, amount **(B)**, episode number **(C)**, and duration **(D)** of each stage in the first 2 h. Data are the means ± SEM (*n* = 8–11). *p < 0.05, **p < 0.01, and ***p < 0.001 compared to vehicle; ^#^ *p* < 0.05 compared to (m)VD-HPα 13.4. Statistics were analyzed by one-way ANOVA and followed by Fisher’s LSD test.

As shown in Figure 3A, compared to vehicle rats, (m)VD-HPα 20.1 and Win55,212-2 rats had a significant decrease in NREM sleep latency (9.8 ± 1.1 and 8.9 ± 1.6 min vs. 15.6 ± 1.6 min, both *p* < 0.01) but not in REM sleep latency. In Figure 3B, the quantitative analysis showed that the amount of NREM sleep in rats injections of 13.4 and 20.1 nmol of (m)VD-HPα and Win55,212-2 increased by 21.6% (*p* < 0.05), 45.3% (*p* < 0.001), and 33.0% (*p* < 0.05), respectively, as compared to that in vehicle rats during the first 2 h post dosing; concomitantly, wakefulness decreased by 11.0% (*p* = 0.079), 26.4% (*p* < 0.01), and 20.2% (*p* < 0.05). Compared to (m)VD-HPα 13.4 rats, (m)VD-HPα 20.1 rats showed an increase in NREM sleep (*p* < 0.05) and a decreased wakefulness (*p* < 0.05, Figure 3B). Moreover, the increase in the NREM sleep in (m)VD-HPα 20.1 rats was due to the increased episode duration, while in Win55,212-2 rats was because of the increased episode number (Figures 3C, D). The wakefulness decreased because of a significant reduction of episode duration in the rats injected with Win55,212-2 or 20.1 nmol of (m)VD-HPα (Figures 3C, D).

### 3.2 Effect of the cannabinoid receptor antagonist on the sleep–wake alterations induced by (m)VD-HPα

**FIGURE 4:**
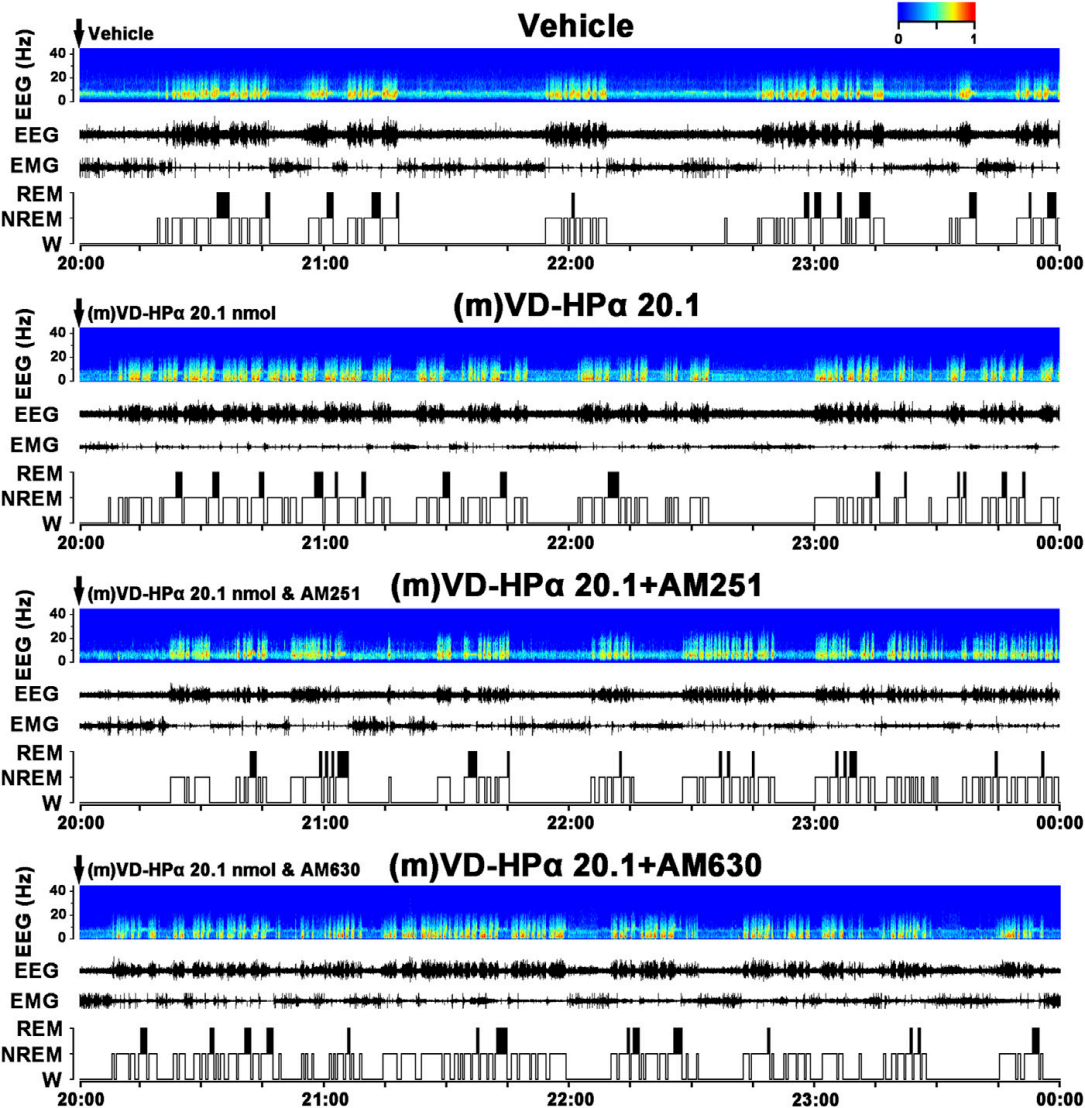
Effects of AM251 and AM630 co-injection with (m)VD-HPα on the EEG and sleep–wake states. Representative 4-h (20:00–00:00) EEG power spectra (frequency 0–30 Hz), EEG, EMG, and hypnograms in rats treated with vehicle, 20.1 nmol of (m)VD-HPα, and 20.1 nmol of (m)VD-HPα with AM251 or AM630 are shown. It should be noted that the rat injection of (m)VD-HPα had a reduced NREM sleep latency and displayed an increase in NREM sleep accompanied by increased cortical 0.5–4.0 Hz activities and suppressed cortical 14.5–30 Hz activities. The rat co-injection of (m)VD-HPα with AM251 [(m)VD-HPα 20.1 + AM251] but not AM630 [(m)VD-HPα 20.1 + AM630] showed a prolonged NREM sleep latency and increased wakefulness accompanied by decreased cortical 0.5–4.0 Hz activities and increased beta activities.

To identify whether the cannabinoid receptor antagonist blocked the effect of (m)VD-HPα, CB_1_ receptor antagonist AM251 (20 nmol, i.c.v.) or CB_2_ receptor antagonist AM630 (20 nmol, i.c.v.) was injected with 20.1 nmol of (m)VD-HPα. As typical examples of the EEG power spectra, EEG, EMG, and the corresponding hypnograms are shown in Figure 4. The rat injection of (m)VD-HPα 20.1 nmol and AM251 ((m)VD-HPα 20.1 + AM251) showed a prolonged NREM sleep latency and an increased wakefulness accompanied by fast low-voltage activity in cortical EEG and a dense EMG activity compared to (m)VD-HPα 20.1 rat (Figure 4). Cortical EEG power spectrum analysis showed that AM251 induced decreased delta and alpha activities and increased beta activities compared to (m)VD-HPα 20.1 rat (Figure 4). However, AM630 did not antagonize the effect of (m)VD-HPα on sleep–wake states and EEG power spectra (Figure 4).

**FIGURE 5:**
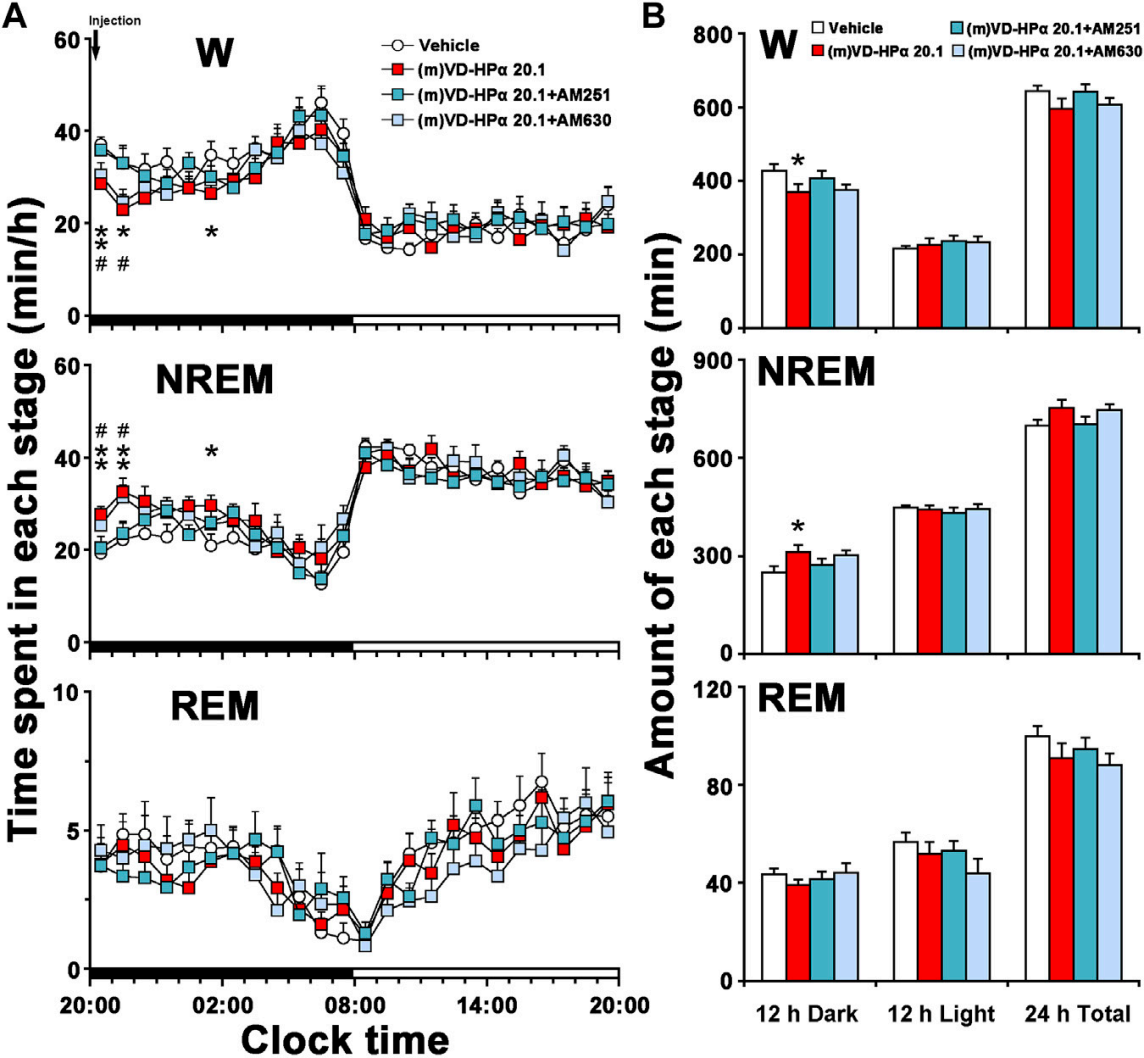
Sleep–wake profiles of rats treated with vehicle, 20.1 nmol of (m)VD-HPα, and 20.1 nmol of (m)VD-HPα with AM251 or AM630. **(A)** Time–course changes in W, NREM, and REM sleep for 24 h [*n* = 9–11, two-way repeated-measures ANOVA followed by the LSD *post hoc* test; W: F_3,35_ = 3.658, *p* = 0.022; NREM: F_3,35_ = 4.325, *p* = 0.011; and REM: F_3,35_ = 1.226, *p* = 0.315; *p < 0.05 and **p < 0.01 compared to vehicle, ^#^ *p* < 0.05 compared to (m)VD-HPα 20.1]. The horizontal filled and open bars indicate the 12-h dark and 12-h light periods, respectively. **(B)** Total time spent in W, NREM, and REM sleep during the 12-h dark and 12-h light phases and 24 h total [*n* = 9–11, one-way ANOVA followed by the LSD *post hoc* test, *p < 0.05, **p < 0.01 compared to vehicle, ^#^ *p* < 0.05 compared to (m)VD-HPα 20.1]. Data are the means ± SEM.

An analysis of the hourly amount of the time spent in each stage over 24 h revealed that AM251 antagonized (m)VD-HPα-induced increase in NREM sleep and decrease in W during 20:00–22:00 h in the dark period (Figure 5A, both *p* < 0.05); however, AM630 did not alter these changes (Figure 5A). A cumulative amount of each stage in 12-h dark and light phases and 24-h total showed that the increased NREM sleep and decreased W induced by (m)VD-HPα in 12-h dark phase were blocked by AM251 (Figure 5B, both *p* < 0.05) but not by AM630 (Figure 5B).

**FIGURE 6:**
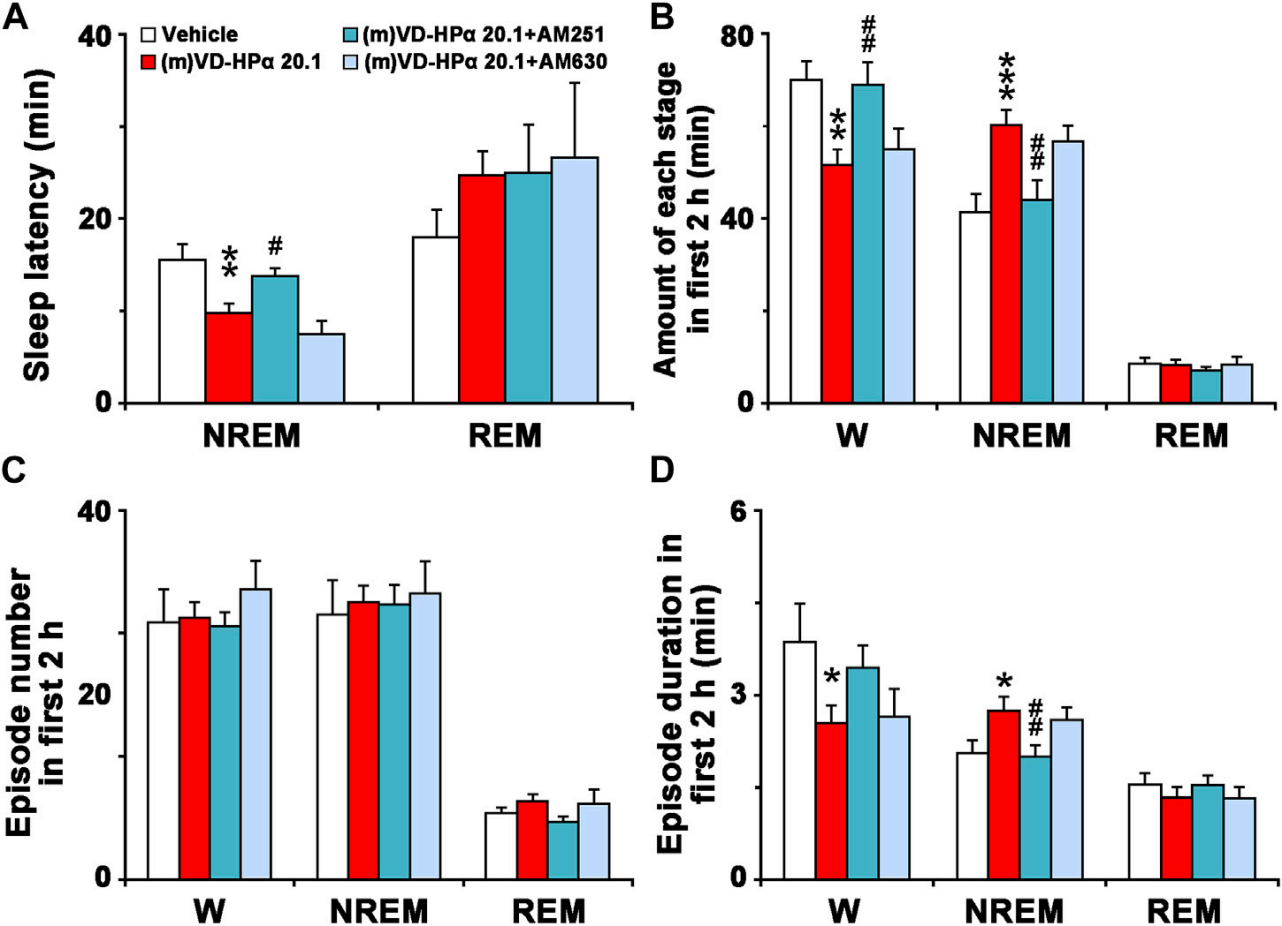
Sleep latency **(A)**, amount **(B)**, episode number **(C)**, and duration **(D)** of each stage in the first 2 h. Data are the means ± SEM (*n* = 9–11). *p < 0.05, **p < 0.01, and ***p < 0.001 compared to vehicle. Statistics were analyzed by one-way ANOVA and followed by Fisher’s LSD test.

As shown in Figure 6A, in comparison with (m)VD-HPα 20.1 rats, (m)VD-HPα 20.1 + AM251 rats had a significant increase in NREM sleep latency (13.8 ± 0.8 min vs. 9.8 ± 1.0 min, *p* < 0.05). In Figure 6B, the quantitative analysis of the first 2 h after dosing showed that the increased amount of NREM sleep induced by (m)VD-HPα was inhibited by AM251 (*p* < 0.01) through reducing episode duration (Figures 6C, D, *p* < 0.01). Concomitantly, the decrease in W caused by (m)VD-HPα was blocked by AM251 (Figure 6B, *p* < 0.01) mainly through increasing episode duration, although not reaching statistical significance (Figures 6C, D, *p* = 0.141). However, AM630 did not antagonize the effect of (m)VD-HPα on NREM sleep latency, episode number, and duration of each stage (Figures 6A–D).

### 3.3 (m)VD-HPα induced c-Fos labeling neurons in the VLPO, LH, TMN, and LC

**FIGURE 7:**
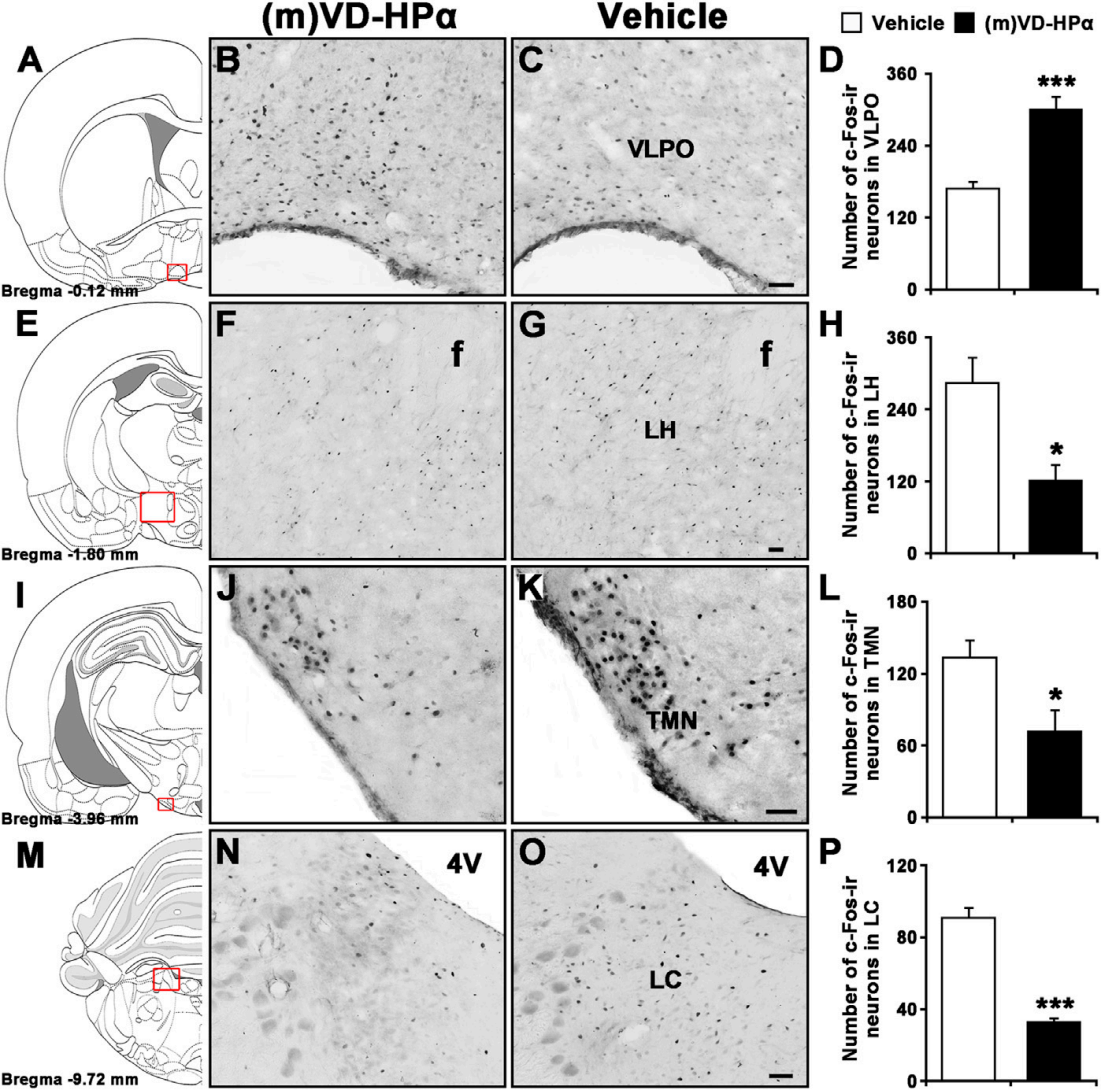
Effects of i.c.v. injection of (m)VD-HPα on Fos immunoreactivity (ir) in the VLPO, LH, TMN, and LC. Schematic drawings show the sections illustrated in **(B)** and **(C)** (Bregma −0.12 mm), **(F)** and **(G)** (Bregma −1.80 mm), **(J)** and **(K)** (Bregma −3.96 mm), and (**N)** and (**O**) (Bregma −9.72 mm) (Paxinos and Watson, 2007). Photomicrographs show Fos-ir neurons (black dots) in the VLPO, LH, TMN, and LC in (m)VD-HPα- and vehicle-treated rats. Histograms show quantitative analysis of the number of For-ir neurons in the VLPO (**D**, t_8_ = 9.873, *p* < 0.001), LH (**H**, t_8_ = 3.270, *p* = 0.011), TMN (**L**, t_8_ = 2.748, *p* = 0.025), and LC (**P**, t_8_ = −5.414, *p* = 0.0006) following (m)VD-HPα (*n* = 5) or vehicle (*n* = 5) i.c.v. injection. Values are means ± SEM. *p < 0.05 and ***p < 0.001. Data were analyzed by unpaired Student’s *t*-test. Bar = 50 μm. Abbreviations: f, fornix; LC, locus coeruleus; LH, lateral hypothalamus; TMN, tuberomammillary nucleus; and VLPO, ventrolateral preoptic nucleus.

Compared to vehicle, the injection of (m)VD-HPα increased the number of Fos-immunoreactive (-ir) neurons in the VLPO (Figures 7A–D, 300.2 ± 21.7 vs. 169.1 ± 10.6; *p* < 0.001) and decreased the number of Fos-ir neurons in the LH (Figures 7E–H, 121.6 ± 25.8 vs. 284.1 ± 42.5; *p* < 0.05), TMN (Figures 7I–L, 71.6 ± 17.6 vs. 133.6 ± 14.1; *p* < 0.05), and LC (Figures 7M–P, 33.1 ± 1.7 vs*.* 90.9 ± 5.6; *p* < 0.001).

## 4 Discussion

The present study demonstrates for the first time that centrally administered peptidic endocannabinoid (m)VD-HPα decreased the NREM sleep latency and promoted NREM sleep through prolonging the mean episode duration. The effects were accompanied by an enhancement of cortical slow activities and a suppression of fast rhythms (Figures 1–3). These effects of (m)VD-HPα (20.1 nmol) on sleep–wake states were similar to that induced by WIN55,212-2 (2.5 nmol) and were significantly blocked by CB_1_ receptor antagonist AM251 but not by CB_2_ receptor antagonist AM630 (Figures 4–6).

These findings are complementary to the earlier opinion that the endocannabinoid system is considered an attractive target for regulating the sleep–wake cycle (Huitron-Resendiz et al., 2004; Prospéro-García et al., 2016). Cannabinoids have been used by humans for many years to alleviate insomnia (Russo and Guy, 2006), and obese patients in clinical trials treated with the CB_1_ receptor antagonist rimonabant commonly reported insomnia and poor sleep quality (Steinberg and Cannon, 2007). Endocannabinoid receptors have been traditionally considered to act through the effects of lipid endocannabinoids (Bushlin et al., 2010; Han et al., 2014). Recently, a series of interesting studies indicated that cannabinoid receptors were recognized by the novel hemopressin-related peptidic endocannabinoid with affinities in the nanomolar range (Heimann et al., 2007; Gomes et al., 2009; Han et al., 2014). In the present study, (m)VD-HPα shortened the NREM sleep latency and promoted NREM sleep during the first 2 h section accompanied by an increase in EEG delta activity, subsequently with a slight increase in the NREM sleep during the dark period (Figures 1, 2); however, NREM and REM sleep were not significantly changed in the light period (Figure 2). Furthermore, the effects of (m)VD-HPα were selectively blocked by CB_1_ receptor antagonist AM251 but not by CB_2_ receptor antagonist AM630 (Figures 4–6), which indicates that (m)VD-HPα promotes NREM sleep through activating the CB_1_ receptor. The CB_1_ receptors are localized primarily on the presynaptic terminals and coupled to Gα_i/o_ (Melis et al., 2004; Tasker et al., 2015); in most cases, stimulation of CB_1_ receptors leads to an inhibition of synaptic transmission in the central nervous systems (Schlicker and Kathmann, 2001). Actually, CB_1_ receptor knock-out mice showed decreased NREM sleep and increased wakefulness (Pava et al., 2014; Silvani et al., 2014), the administration of the CB_1_ receptor antagonist induced a dose-dependently increase in wakefulness (Santucci et al., 1996), and in the present study, the traditional CB_1_ receptor agonist WIN55,212-2, used as positive control, also increased NREM sleep (Figure 2). These evidence support that the endocannabinoid system plays a vital role in regulating the sleep–wake cycle, and the activation of the CB_1_ receptor probably promotes NREM sleep. We note that the biphasic effect of cannabis on sleep has existed (Babson et al., 2017), and commonly, the endocannabinoid has been considered to increase NREM sleep (Rueda-Orozco et al., 2010; Babson et al., 2017); however, phytocannabinoid, such as cannabidiol, has been shown differential effects on sleep based on dose (Chagas et al., 2013; Babson et al., 2017), low-dose cannabidiol has been associated with increased wakefulness (Nicholson et al., 2004; Zuardi, 2008), while high dosage has been proved to increase total sleep time (Carlini and Cunha, 1981). To further study the effect of different dosages of (m)VD-HPα on NREM sleep, according to Han et al. (2014), 6.7, 13.4, or 20.1 nmol of (m)VD-HPα was administrated into the intracerebral ventricle. Our results showed that (m)VD-HPα produced a dose-related increase in NREM sleep (Figures 1, 2), and the analogous dose-dependent phenomena were reported in the previous experiment that (m)VD-HPα exerted antinociceptive effects (Han et al., 2014). We note that the CB_1_ receptor is involved in REM sleep (Pérez-Morales et al., 2013); however, in the present study, (m)VD-HPα did not induce significant changes in REM sleep, the inconsistent results might be due to the type of cannabinoids, dosage, and route of administration. Collectively, our data imply that (m)VD-HPα dose dependently promotes NREM sleep through activation of the CB_1_ receptor.

Consistent with the previous study, we found that the increased NREM sleep induced by (m)VD-HPα was primarily due to increased NREM bout length (Figure 3), and these effects were selectively blocked by CB_1_ receptor antagonist AM251 (Figure 6). The prolonging duration of wake and sleep bouts commonly implies a stable sleep–wake state (Saper et al., 2010), and several research studies have found that direct activation of the CB_1_ receptor with CP47,497 augmented the time spent in NREM due to increased bout length (Pava et al., 2016). In addition, the CB_1_ receptor knock-out mice displayed fragmented NREM sleep (Pava et al., 2014; Silvani et al., 2014) to further support for the role of the endocannabinoid system in regulating NREM stability. We note that fragmentary sleep is a notable sign of anxiety disorder (Jakubcakova et al., 2012; Xie et al., 2018), and lots of anxiolytics extend the duration of NREM bout (Monti and Monti, 2000). Interestingly, recent studies showed that hemopressin, an inverse agonist of the CB_1_ receptor, induced anxiogenic-like effects (Fogaça et al., 2015); thus, we hypothesized that the new peptidic endocannabinoid (m)VD-HPα might counteract the anxiety-like behavior and sleep disturbances. These evidence, together with our findings, demonstrate that (m)VD-HPα stabilizes the NREM sleep state through activation of the CB_1_ receptor and may have therapeutic potential for the treatment of insomnia, especially comorbidity with anxiety.

To identify the target neurons through which (m)VD-Hpα promotes NREM sleep, we labeled activated neurons by staining c-Fos, one product of the immediate early gene that is expressed in association with neuronal activation (Zhao et al., 2012; Shao et al., 2016; Xie et al., 2018). The results show that (m)VD-HPα increased the Fos-ir neurons in VLPO, a necessary part of the brain circuitry that produces sleep, suggesting the involvement of the GABAergic system in the action of (m)VD-HPα in the sleep–wake cycle. Furthermore, the number of Fos-ir neurons in LH, LC, and TMN, the regions related to the regulation of wakefulness, was reduced after (m)VD-HPα administration. We note that the CB_1_ receptor is expressed in many brain regions, especially, its distribution in the pons and hypothalamus is of fundamental importance here (Melis et al., 2004; Chagas et al., 2013; Tasker et al., 2015), given that the action of (m)VD-HPα on these receptors might inactivate orexinergic, noradrenergic, and histaminergic neurons to increase NREM sleep. Importantly, pioneer studies showed that CB_1_ receptors were primarily localized on the pre-synaptic terminals of glutamatergic and GABAergic synapses (Hirasawa et al., 2004; Tasker et al., 2015), where they reduce the release probabilities of glutamate and GABA and modulate excitatory and inhibitory neurotransmission (Tasker et al., 2015). These opinions may explain the phenomenon that (m)VD-HPα increased the number of Fos-ir neurons in the VLPO and decreased that in LH, LC, and TMN through the activation of the CB_1_ receptor. However, as one limitation, we did not identify whether (m)VD-HPα directly activates the GABAergic neurons in the VLPO or directly inhibits the orexinergic, noradrenergic, and histaminergic neurons in LH, LC, and TMN, respectively, in the present study. Nevertheless, our findings suggest that (m)VD-HPα, the peptidic endocannabinoid, promotes NREM sleep via the CB_1_ cannabinoid receptor to probably inactivate the neurons in LH, LC, and TMN and activate the neurons in the VLPO.

## 5 Conclusion

Central administration of peptidic endocannabinoid (m)VD-HPα significantly enhances NREM sleep and reduces wakefulness. (m)VD-HPα-induced NREM sleep enhancement was due to the extended episode duration instead of the episode number. These effects are significantly blocked by CB_1_ receptor antagonist AM251 but not by CB_2_ receptor antagonist AM630. (m)VD-HPα markedly increases the number of Fos-ir neurons in the VLPO and decreases that in the LC, LH, and TMN. These results indicate that (m)VD-HPα promotes and stabilizes NREM sleep via the CB_1_ cannabinoid receptor to probably activate VLPO GABAergic neurons and inactivate the LH orexinergic, LC noradrenergic, and TMN histaminergic neurons.

## Data Availability

The raw data supporting the conclusion of this article will be made available by the authors, without undue reservation.
